# Supplementary material for: State-recycling and time-resolved imaging in topological photonic lattices
Source: Nat Commun. 2018 Oct 11;9:4209. doi: 10.1038/s41467-018-06723-y (PMC6181942; doi:10.1038/s41467-018-06723-y)
Supplement: Supplementary file 1 — Supplementary Information [file 41467_2018_6723_MOESM1_ESM.pdf]

Supplementary Information:

State-recycling and time-resolved imaging in topological photonic lattices

Mukherjee et al.

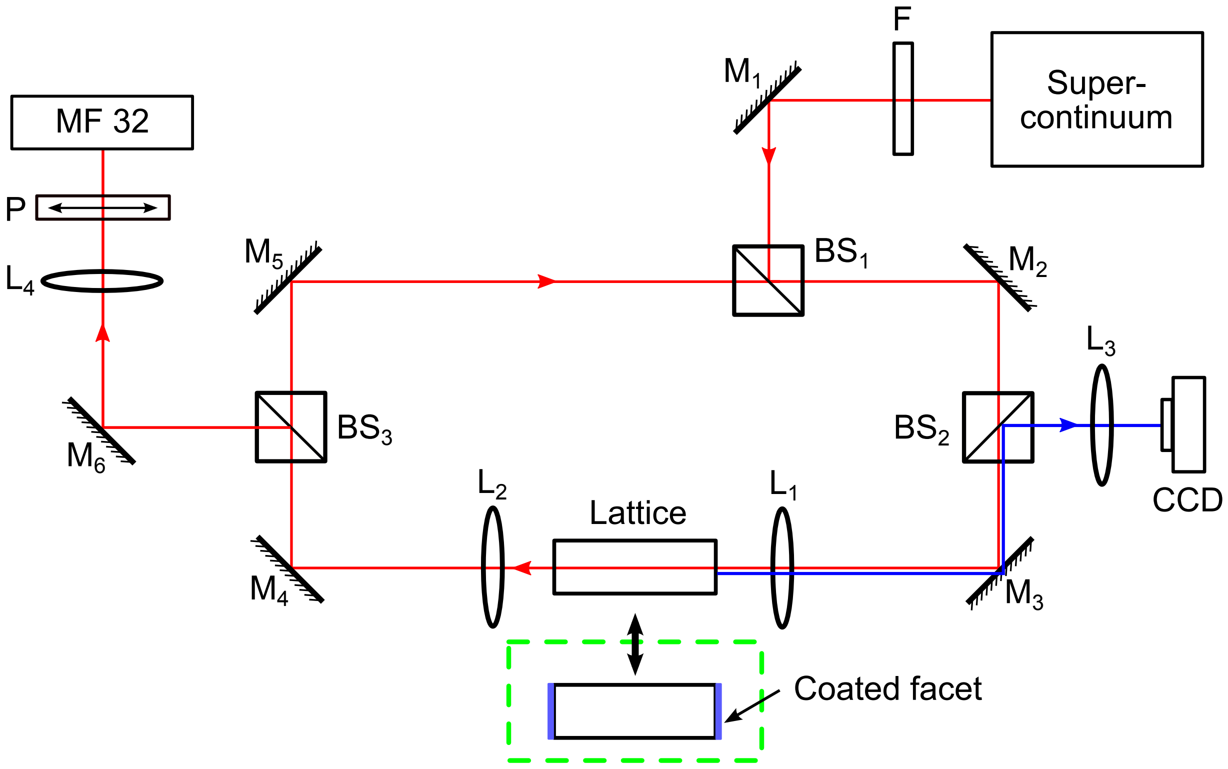

Supplementary Figure 1: **Experimental setup.** Here, $L_{1,5}$ are convex lenses, M_{1-6} are silver-coated mirrors, F is a bandpass filter at 780 ± 5 nm wavelength, BS_{1-3} are beam splitters and P is a polariser. M_{2-5} form the ring cavity. For precise imaging, the input facet of the lattice is imaged on a CCD camera (see the blue line) to observe the lattice sites, input state, and the state after the first pass. To perform the state-recycling using a linear cavity, the lattice is replaced by another lattice with silver-coated facets (shown in the green-dashed inset), BS_3 is replaced by a mirror to reflect the output state onto the Megaframe (MF32) and F is replaced by another bandpass filter as required for the experiment.

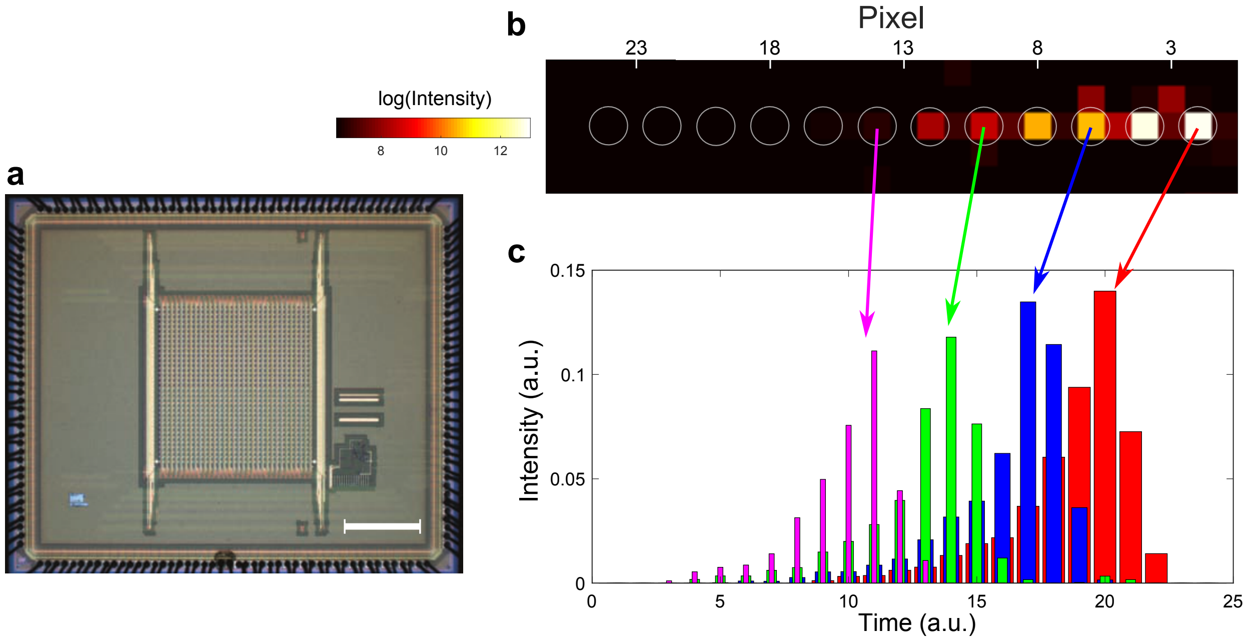

Supplementary Figure 2: **Data processing. a,** Optical micrograph of the Megaframe (MF32) camera consisting of 32×32 single-photon avalanche detector array, scale-bar: 800 μm. **b,** Spatial information i.e. intensity distribution summed over four and a half round trips for the driven one-dimensional lattice presented in Figure 1 in the main text. To reduce spatial overlap of light intensity from two consecutive lattice sites, optical modes were imaged onto alternative pixels of the MF32 (represented by white circles). Each pixel contains temporal information. **c,** Temporal intensity profiles for four pixels indicated by the coloured arrows. These peaks are normalised such that the total detected optical power for each round trip is unity. Here, the time-step (horizontal axis) is ~ 53 ps.

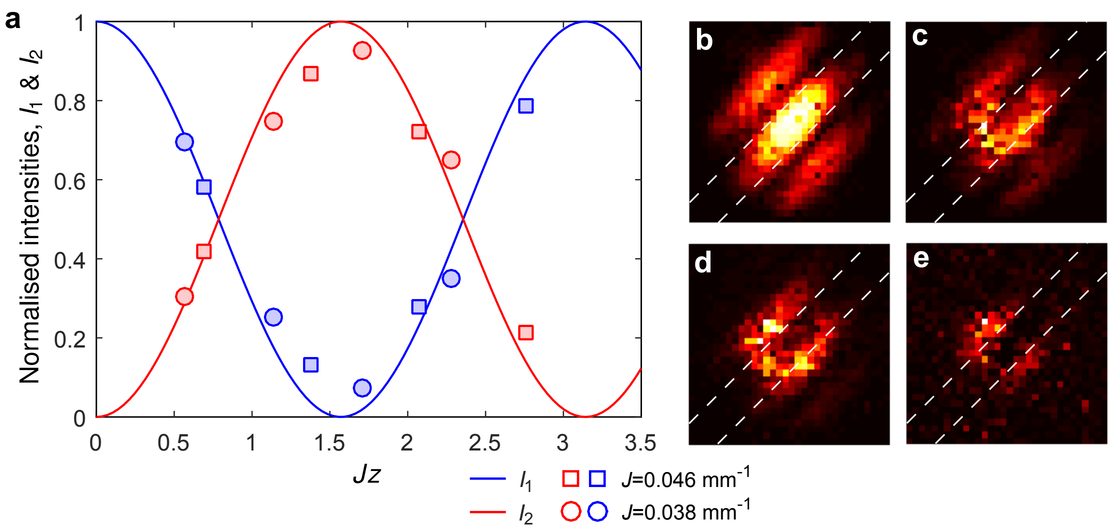

Supplementary Figure 3: **Intensity and phase recycling in the ring cavity. a,** A directional coupler (i.e. two evanescently coupled identical waveguides) was placed inside the ring cavity, light was initially launched at waveguide-1 and the evolution of optical fields was measured in a time-resolved manner. The solid lines indicate the expected variation of light intensity at waveguide-1 (blue) and waveguide-2 (red) as a function of Jz. Two sets of experiments were performed using optical pulse trains at 780 nm (square) and 750 nm wavelength (circle) which corresponds to two sets of tunnelling strengths, $J = 0.046$ and 0.038 per mm, respectively. **b-e,** Time-resolved interference experiment at 750 nm wavelength. **b-e** show interference fringes (at four consecutive round trips) generated by allowing the modes at the output of the coupler to interfere on the single-photon avalanche detector array in the far-field. The fringes are rotated at $45°$ because the coupling axis between the waveguides was orientated at that angle with respect to the vertical axis. The dashed lines are guides to the eye. The π phase shift observed in **d** and **e** compared to **b** and **c** is a characteristic of a directional coupler – after the full transfer of light, i.e. $Jz > \pi/2$, the relative phase between the optical modes of the waveguides exhibit a phase shift of π. These experiments prove that both phase and intensity of optical fields is recycled in the ring cavity scheme.

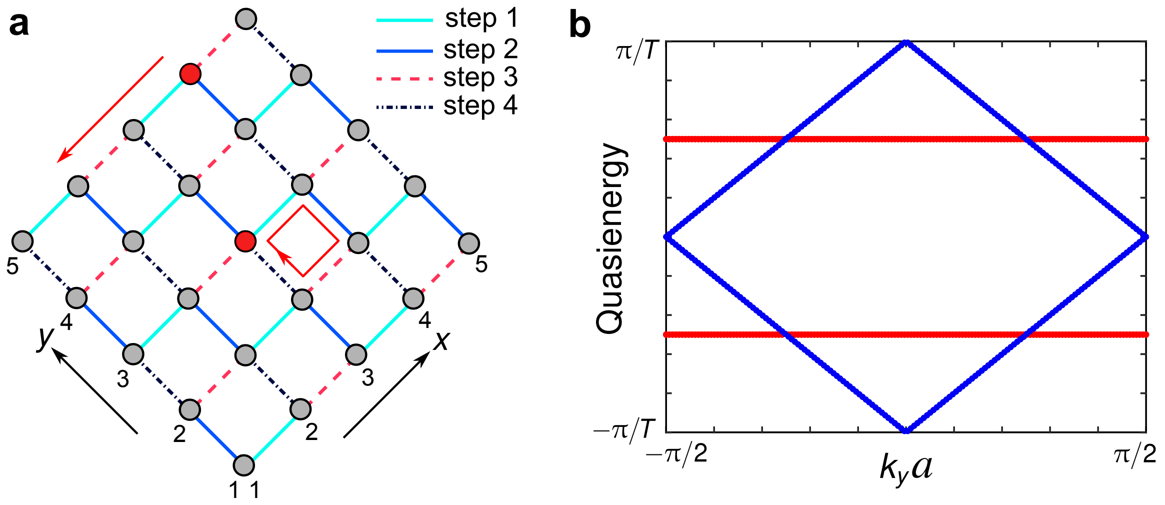

Supplementary Figure 4: **Driving protocol and band structure of a driven photonic lattice.** **a,** A slowly-driven square lattice with nearest-neighbour couplings (J_{1-4} corresponding to step 1-4), which are varied in a spatially homogeneous and time-periodic manner. For $J_{1-4}T/4 = \pi/2$, anomalous topological edge modes coexist with a localised bulk. In this case, the Floquet bulk bands are degenerate at zero quasienergy. **b,** Floquet quasienergy spectrum for $J_1T/4 = 0$ and $J_{2,3,4}T/4 = \pi/2$. Here, the bulk bands (shown in red) with zero Chern number are gapped while the winding numbers associated with both the energy gaps (centred on 0 and π/T) are one.

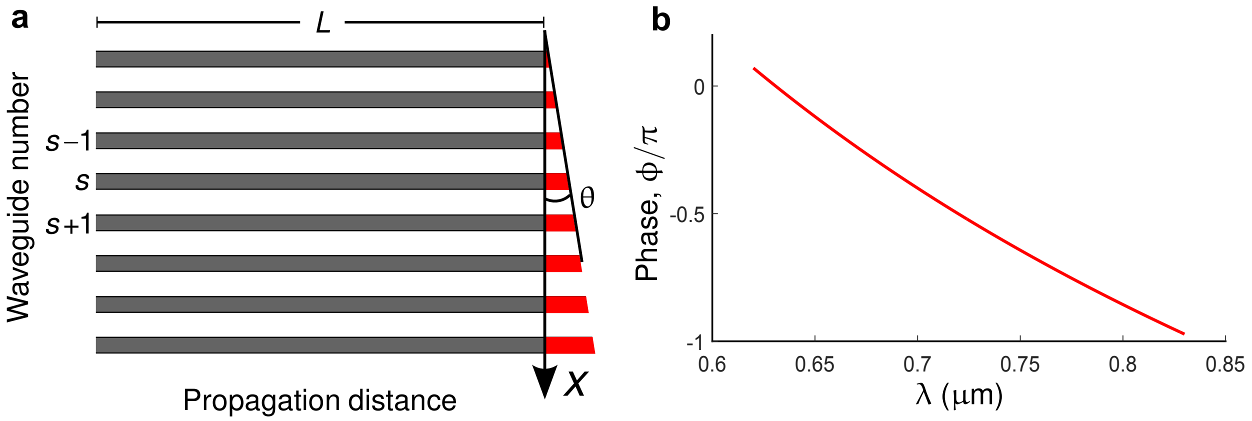

Supplementary Figure 5: **Floquet engineering of an electric field. a,** The output facet of the photonic lattice is polished at a small angle θ with respect to x axis, to realize an instantaneous pulsed electric field which exists for an infinitesimally short duration of time. **b,** For a specific value of $\theta = 2^{\circ}$, the inter-waveguide phase shift (ϕ) can be tuned by varying the wavelength of incident light, λ.

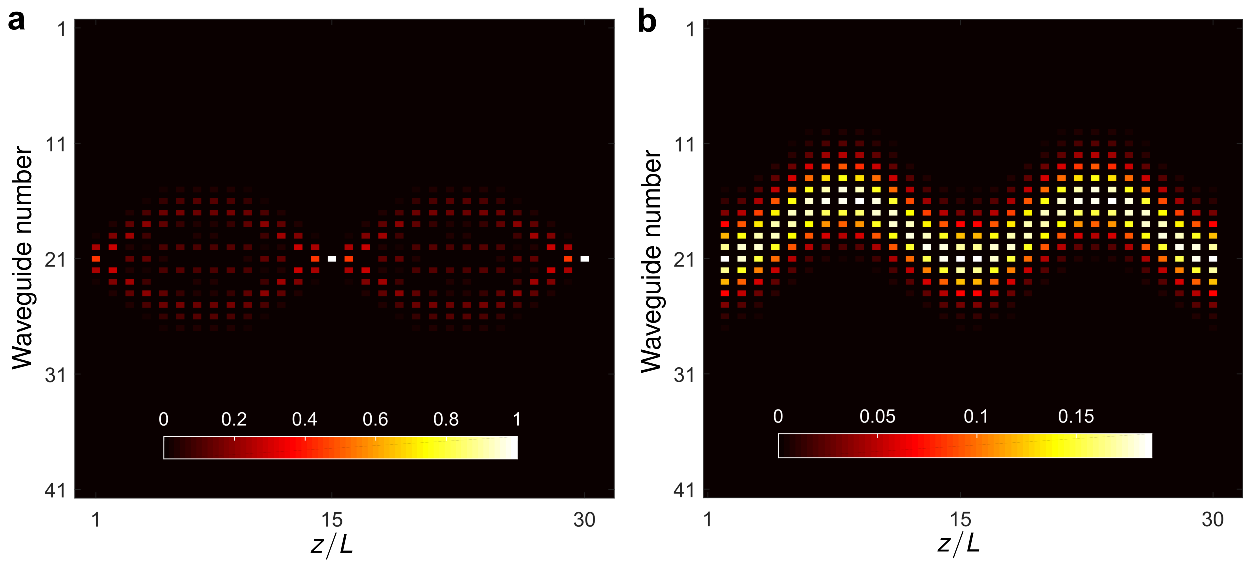

Supplementary Figure 6: **Time-periodic pulsed electric field along the lattice axis and Bloch oscillations.** A finite photonic lattice with 41 waveguides and $\phi = \pi/7.5$ is considered. Here $J = 0.02$ mm^{-1} and $L = 30$ mm. **a,** Numerically calculated evolution of optical intensity when only the 21-st waveguide is excited initially; here a breathing motion of the intensity pattern is observed. **b,** In this case a broad initial state (in real space) is considered and the oscillation of the wave-packet's centre of mass is observed.

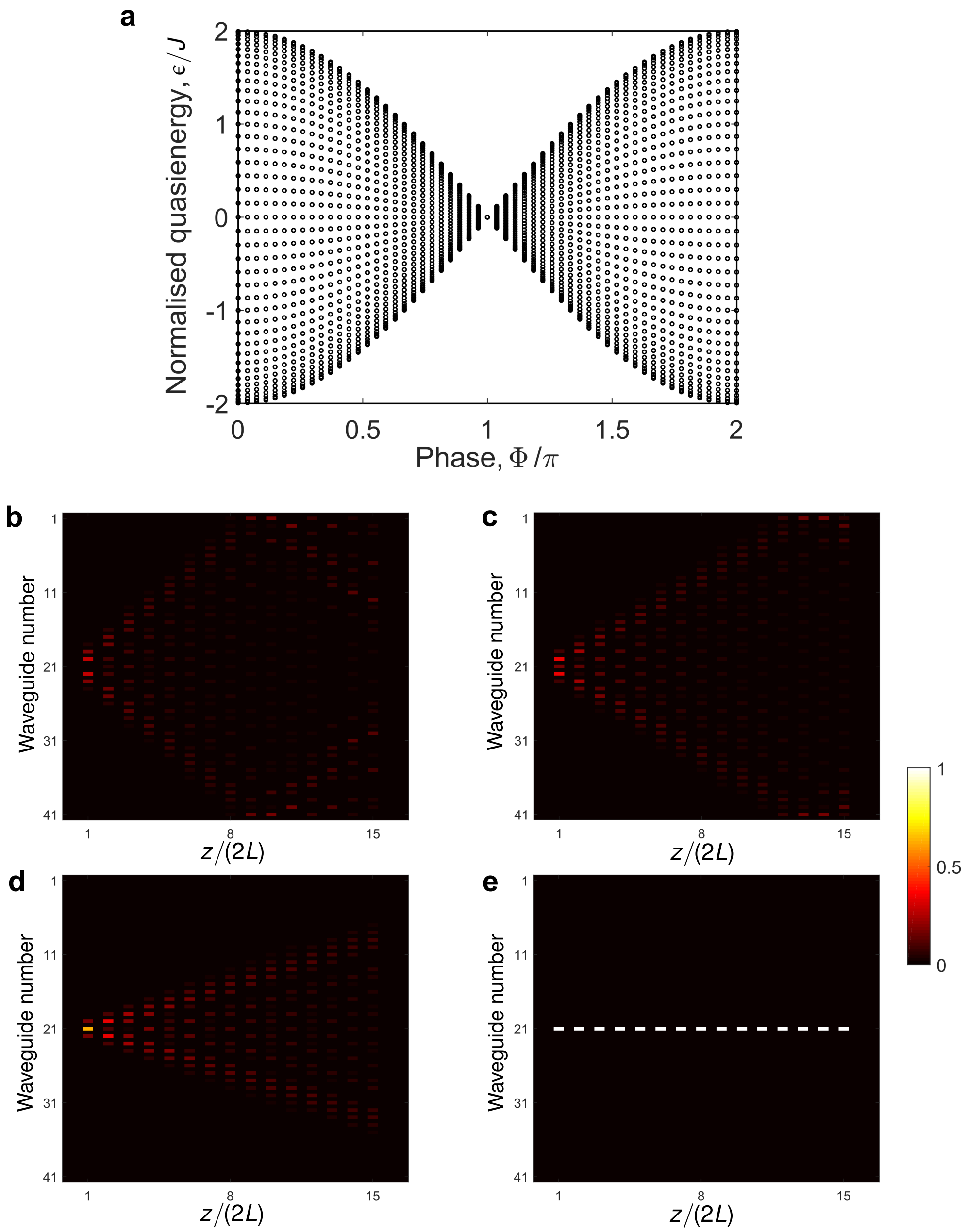

Supplementary Figure 7: **Floquet engineering of alternating pulsed electric field. a,** Here we consider a straight photonic lattice inside a linear cavity with both facets polished at equal angles, θ. In this case, alternating electric field pulses are realised. Floquet quasienergy spectrum as a function of the phase shift between adjacent sites ($2\phi = \Phi$). The band collapses at $\Phi = \pi$ exhibiting destruction of tunnelling. **b-e,** Numerically calculated evolution of the intensity distributions for $\Phi = 0, \pi/2, 3\pi/4$ and π, respectively.

Supplementary Note 1: A one-dimensional driven lattice and the Dirac Hamiltonian

In this section, we present the driving protocol related to the 1D lattice illustrated in Figure 1b (in the main text) as well as its photonic implementation (Figure 1c). Let us first consider a general situation, a 1D tight-binding lattice with staggered hopping amplitudes ($J_{1,2}$). In the static case, the k-space Hamiltonian can be written as

$$\hat{H}_k = (J_1 + J_2)\cos(kd)\hat{\sigma}_x - (J_1 - J_2)\sin(kd)\hat{\sigma}_y, \tag{1}$$

where d is the inter-site separation, $\hat{\sigma}_{x,y,z}$ are Pauli matrices and the Brillouin zone spans $0 \leq k \leq \pi/d$. Now consider that $J_{1,2}$ are varying in a time periodic manner with a period T. The driving protocol is the following:

$$J_1 = 0 \text{ and } J_2 = \pi/T \quad \text{for } 0 \leq t \leq T/2; \tag{2}$$

$$J_1 = \pi/T \text{ and } J_2 = 0 \quad \text{for } T/2 \leq t \leq T. \tag{3}$$

Note that the probability for a particle to hop to its nearest site is unity when the tunnelling/hopping is allowed. The Floquet operator and the effective Hamiltonian for this two-step driving protocol can be written as [1]

$$\begin{aligned}\hat{U}(T) &= e^{-i\hat{H}_2 T/2} e^{-i\hat{H}_1 T/2} \\ &= \exp[-i(-2kd\hat{\sigma}_z + \pi\hat{1})] = e^{-i\hat{H}_{\text{eff}} T},\end{aligned} \tag{4}$$

$$\hat{H}_{\text{eff}} = v_D k \hat{\sigma}_z + \text{cst}, \qquad v_D = -2d/T, \tag{5}$$

where $\hat{H}_{1,2}$ are the Hamiltonians for the two driving steps and $\hat{1}$ is a 2×2 identity matrix. Equation (5) is the 1D Dirac Hamiltonian, which describes pseudo-relativistic particles in linearly-

dispersive bands, with a speed of light $v_D = 2d/T$. The Floquet spectrum associated with Supplementary Equation (5) is shown in Figure 1d (main text).

Now let us discuss the photonic implementation of the driving protocol in Supplementary Equation (2) and (3). To perform the state-recycling using a linear cavity, we consider a driven photonic lattice of length L (as shown in Figure 1c) where each bond is a $50:50$ directional coupler. Note that light travels along the $+z$ direction for the first half of the complete driving period ($T \equiv 2L$) and then almost 90% of light reflects back and travels along the $-z$ direction. For the time-correlated single photon counting (TCSPC) measurement, the transmitted light is imaged onto the SPAD array. The four-step driving protocol for the photonic lattice can be written as follows:

Light propagation along $+z$ where

$$J_1 = 0 \text{ and } J_2 = \pi/(2L) \quad \text{for } 0 \leq z \leq L/2; \tag{6}$$

$$J_1 = \pi/(2L) \text{ and } J_2 = 0 \quad \text{for } L/2 \leq z \leq L; \tag{7}$$

and after reflection (i.e light propagation along $-z$)

$$J_1 = \pi/(2L) \text{ and } J_2 = 0 \quad \text{for } L \leq z \leq L/2; \tag{8}$$

$$J_1 = 0 \text{ and } J_2 = \pi/(2L) \quad \text{for } L/2 \leq z \leq 0. \tag{9}$$

Note that the propagation distance is the analogous time ($z \leftrightarrow t$). The Floquet operator for the four-step driving is given by

$$\begin{aligned} \hat{U}(T) &= \mathcal{T} \textstyle\sum_n e^{-i\hat{H}_n T/4} \\ &= \exp(-i[2kd(\sin(kd)\hat{\sigma}_x - \cos(kd)\hat{\sigma}_y) + \pi\hat{1}]), \end{aligned} \tag{10}$$

where $\mathcal{T}$ indicates the time ordering and $n=1,2,3,4$. The effective Hamiltonian then becomes

$$\hat{H}_{\text{eff}} = (2d/T)k(\sin(kd)\hat{\sigma}_x - \cos(kd)\hat{\sigma}_y) + (\pi/T)\hat{1}. \tag{11}$$

It can be shown that the effective Hamiltonian in Supplementary Equation (11) is equivalent to the Dirac Hamiltonian in Supplementary Equation (5), up to a unitary transformation. The same result could be simply obtained by noting that the sequence (6)-(9) is equivalent to that of (2)-(3), but in a different time frame.

Supplementary Note 2: Brief description of anomalous topological edge modes

Topological band theory of a static system can be extended to a time periodic system using Floquet theory. In the presence of a high frequency driving, i.e. driving frequency $\gg$ inter-site coupling strength, the topology of the system can be predicted by the usual topological invariants (e.g. Chern numbers) that are used for a static system [2]. However, away from the limit of high frequency driving, i.e. driving frequency $\sim$ inter-site coupling strength, topologically protected edge modes can be observed even if the Chern numbers associated with all the bulk bands are zero. Such anomalous topological edge modes can be characterised by a distinct topological invariant known as the winding number [3].

Here we consider a square lattice [4] with nearest neighbour coupling J_{1-4} which are varying spatially and in a time periodic manner as shown in Supplementary Figure 4a. Imagine that the driving period, T, is equally split into four steps and $J_i T/4 = \Lambda_i$. The Floquet operator for this driven lat-

tice is given by

$$U(T) = e^{-iH_4T/4} e^{-iH_3T/4} e^{-iH_2T/4} e^{-iH_1T/4}, \tag{12}$$

where the Hamiltonian, $\hat{H}_n$ $(n = 1, 2, 3, 4)$, is piece-wise constant in time within the interval $(n - 1)T/4 \leq t \leq nT/4$, see Supplementary Figure 4a. Now consider that the first bond [step 1 in Supplementary Figure 4a] is always off i.e. $\Lambda_1 = 0$ and for other three bond $\Lambda_{2-3} = \pi/2$. For this driving protocol, the Floquet spectrum consists of two non-degenerate flat bands with zero Chern number, see Supplementary Figure 4b. Importantly, as the bulk bands are well separated, small disorder (less than the energy gap between the bulk bands) cannot close the energy gap. The winding numbers associated with both the energy gaps (centred on 0 and π/T) are one. It should be highlighted that the magnitude of the group velocity of the chiral edge modes along the top-right edge of the lattice (i.e. along y direction) is twice of that along the top-left (i.e. along x direction).

Supplementary Note 3: Floquet engineering of an additional electric field

In this section, we show how the state-recycling technique can allow for the engineering of additional fields, which can then act on top of the (effective) Hamiltonian associated with the photonic lattice. Here, we illustrate this concept by showing how an effective electric field can be simply generated by modifying the very end of the lattice (which corresponds to a time-periodic pulsed electric field). Before describing this method, let us point out that such pulsed fields could also be digitally engineered, by changing the output (recycled) state according to a well-defined unitary operator.

Let us consider a straight 1D photonic lattice with inter-waveguide separation, d and nearest-neighbour coupling, J. In the grey section illustrated in Supplementary Figure 5a, the optical fields travel and exhibit discrete diffraction for a length L. The output facet of the lattice is polished at a small angle (θ), hence the inter-waveguide light transfer in the red section can be ignored for an experimentally realizable finite array. In this red section, the optical mode at the s-th waveguide will acquire an additional phase, $\phi_s = s(2\pi d n_{\text{eff}}/\lambda)\tan(\theta) = s\phi$ where λ is the wavelength of light, n_{eff} is the effective modal refractive index and ϕ is the inter-waveguide phase shift. It should be noted that this phase shift along the array, which is linear in s, is analogous to an external electric field applied instantaneously for an infinitesimally short duration of time; formally, this corresponds to acting on a state with the unitary operator $e^{-i\tau E\hat{x}}$, where τ is the effective pulse duration, $E = \phi/\tau$ is the effective electric field strength, and where $\hat{x}$ denotes the position operator on the lattice. Supplementary Figure 5b shows the variation of ϕ as a function of wavelength, providing an extra degree of freedom to fine-tune the strength and direction of the electric field. When this lattice is placed inside a ring cavity, the complete driving period, $T = L$, consists of the following two steps:

(1) time evolution determined by the coupling strength, J for $0 \leq z \leq L$;

(2) tunnelling is frozen and an analogous static field is applied at $z = L$.

Formally, the time-evolution operator over each period T can be written in the two-step form,

$$\hat{U}(T) = e^{i(\phi/d)\hat{x}} \times e^{-iT\hat{H}_0}, \tag{13}$$

where $\hat{x} = d\sum_s s|s\rangle\langle s|$ is the position operator on the lattice and $\hat{H}_0 = J\sum_s |s+1\rangle\langle s| + \text{h.c.}$ is the hopping Hamiltonian with amplitude J. For weak synthetic electric fields, $\phi \ll 1$, this

time-evolution operator can be simplified according to the Trotter formula,

$$\hat{U}(T) \approx e^{-iT\left(\hat{H}_0 - \phi/(dT)\,\hat{x}\right)}, \tag{14}$$

which describes the motion of a particle hopping on a lattice in the presence of an effective electric field $E = \phi/dT$. We note that a similar form can be obtained in the large-field regime $\phi \sim 1$, using the full Baker-Campbell-Hausdorff formula.

For the purpose of numerical calculations, a photonic lattice with 41 waveguides and $\phi = \pi/7.5$ is considered. By solving the Schrödinger equation associated with the full Floquet (effective) Hamiltonian, the time evolution of the input state is obtained for two specific input states, see Supplementary Figure 6. Supplementary Figure 6a corresponds to the input state localised at the 21-st waveguide whereas for Supplementary Figure 6b, seventeen waveguides were initially excited with a Gaussian intensity pattern. In both cases, the characteristics of Bloch oscillations are observed– breathing motion of the intensity pattern for the first case and the oscillation of the wave-packet's centre of mass for the second one.

Now let us consider a different scheme generating alternating electric field pulses, which can be realised using a straight photonic lattice inside a linear cavity with both facets polished at equal angles, θ. In this case, the complete driving period, $T = 2L$, can be split into the following steps:

(1) time evolution determined by the coupling strength, J for $0 \leq z \leq L$;

(2) tunnelling is frozen and an analogous static field (characterised by 2ϕ) is applied at $z = L$;

(3) time evolution determined by the coupling strength, J for $L \leq z \leq 0$;

(4) tunnelling is frozen and an analogous static field (characterised by -2ϕ) is applied at $z = 0$.

Formally, the time-evolution operator over each period T can be written in the four-step form,

$$\hat{U}(T) = e^{-i(2\phi/d)\hat{x}} \times e^{-i(T/2)\hat{H}_0} \times e^{i(2\phi/d)\hat{x}} \times e^{-i(T/2)\hat{H}_0}, \tag{15}$$

where we used the same notations as above. Using the following expression [1],

$$e^{i\alpha\hat{x}}\hat{H}_0 e^{-i\alpha\hat{x}} = \hat{H}_0\cos(\alpha) - \hat{H}_1\sin(\alpha), \tag{16}$$

where $\hat{H}_1 = -iJ\sum_s(|s+1\rangle\langle s| - \text{h.c.})$, and noting that $[\hat{H}_1, \hat{H}_0] = 0$, we finally obtain an exact form for the Floquet operator in Supplementary Equation (15) with

$$\hat{U}(T) = e^{-iT\cos(\phi)\hat{H}_0}, \tag{17}$$

which indicates that the pulsed and alternating electric field simply renormalizes the hopping amplitude $J \to J\cos(\phi)$. We note that the effective Hamiltonian appearing in Supplementary Equation (17), namely, $\hat{H}_{\text{eff}} = \cos(\phi)\hat{H}_0$, is valid for any ϕ. In particular, for $\phi = \pi/2$, we find that the Floquet operator is trivial, $\hat{U}(T) = \hat{1}$, which indicates that the hopping is effectively annihilated by the pulsed electric field.

It should be mentioned that this particular four-step driving protocol can also be realised by fabricating the photonic lattice such that the waveguide axes are tilted at an angle with respect to the length of the glass sample. In that case, both facets will be at equal angles with respect to the waveguide axes without the requirement of angle polishing. For this driving protocol, the Floquet quasienergy spectrum as a function of the phase shift between adjacent sites ($2\phi = \Phi$) is presented in Supplementary Figure 7a. As expected, the band indeed collapses at $\Phi = \pi$, indicating destruction of tunnelling. Supplementary Figure 7b-e show numerically calculated evolution of the intensity distributions for $\Phi = 0, \pi/2, 3\pi/4$ and π respectively.

Supplementary Note 4: Discrete diffraction in the presence of a synthetic electric field

In this section, we present experimental details and the driving protocol related to the quasi-real time-resolved imaging of discrete diffraction in the presence of a synthetic electric field; see Figure 2 in the main text. Using ultrafast laser inscription, a 1D straight photonic lattice consisting of twenty coupled single-mode waveguides was fabricated. Both facets of the glass substrate containing the lattice were polished and silver-coated to form a linear cavity. The input and output facet angles with respect to the lattice axis (x) were measured to be $\theta_{1,2} \approx \pm 0.1^{\circ}$, respectively. These small angles at the facets of the substrate (inset in Fig. 2e, main text) cause a linear phase shift along the lattice, which effectively produces a time-periodic (pulsed) synthetic electric field. In contrast to our previous discussion in Supplementary Equation (15), here the angles have opposite signs, which means that the associated synthetic pulsed electric fields will be along the same direction. In this experiment, the total driving period, $T = 2L$, consists of four driving steps:

(1) tunnelling is frozen and an analogous static field (characterised by $+2\phi_1$) is applied at $z = 0$;

(2) time evolution determined by the coupling strength, J for $0 \leq z \leq L$;

(3) tunnelling is frozen and an analogous static field (characterised by $+2\phi_2$) is applied at $z = L$;

(4) time evolution determined by the coupling strength, J for $L \leq z \leq 0$;

Formally, the time-evolution operator over each period T can be written in the four-step form,

$$\hat{U}(T) = e^{-i(T/2)\hat{H}_0} \times e^{i(2\phi_2/d)\hat{x}} \times e^{-i(T/2)\hat{H}_0} \times e^{i(2\phi_1/d)\hat{x}} \tag{18}$$

$$\approx e^{-iT\left(\hat{H}_0 - 2(\phi_1+\phi_2)/(dT)\,\hat{x}\right)}, \tag{19}$$

where $\hat{H}_0$ describes the hopping in the 1D array, see Supplementary Equation (14). Supplementary Equation (19) describes the motion of a particle hopping on a lattice in the presence of an effective electric field $E = 2(\phi_1 + \phi_2)/(dT)$. In our experiment, the tunnelling strength $J = 0.029$ mm^{-1} and the expected inter-waveguide phase shifts are $\phi_{1,2} \approx \pi/12$ which means that the associated Bloch period is $\approx 12L = 360$ mm. In Figure 2 (main text), we probed the dynamics up to 210 mm which is approximately half of this period.

In conclusion, the state-recycling technique introduced in this work indeed allows one to engineer synthetic electric fields, which effectively act on top of the dynamics associated with the engineered photonic lattice. For constant electric field [Supplementary Equation (14)], this could be used to perform transport experiments in view of probing response functions (e.g. the conductivity tensor) or geometric effects through Bloch oscillations [5]. For alternating electric fields, this could be used to control the properties (e.g. tunnelling) of the engineered photonic lattice.

Supplementary References

1. Goldman, N. & Dalibard, J. Periodically driven quantum systems: effective Hamiltonians and engineered gauge fields. *Phys. Rev. X* **4**, 031027 (2014).

2. Hasan, M. Z. & Kane, C. L. Colloquium: topological insulators. *Rev. Mod. Phys.* **82**, 3045 (2010).

3. Rudner, M. S., Lindner, N. H., Berg, E. & Levin, M. Anomalous edge states and the bulk-edge correspondence for periodically driven two-dimensional systems. *Phys. Rev. X* **3**, 031005

(2013).

4. Mukherjee, S. *et al.* Experimental observation of anomalous topological edge modes in a slowly driven photonic lattice. *Nat. Commun.* **8**, 13918 (2017).

5. Wimmer, M., Price, H. M., Carusotto, I. & Peschel, U. Experimental measurement of the Berry curvature from anomalous transport. *Nat. Phys.* **13**, 545–550 (2017).
